# Associations of Genetic Variation in Glyceraldehyde 3-Phosphate Dehydrogenase Gene with Noise-Induced Hearing Loss in a Chinese Population: A Case-Control Study

**DOI:** 10.3390/ijerph17082899

**Published:** 2020-04-22

**Authors:** Liu Wan, Boshen Wang, Juan Zhang, Baoli Zhu, Yuepu Pu

**Affiliations:** 1 Key Laboratory of Environmental Medicine Engineering, Ministry of Education, School of Public Health, Southeast University, Nanjing 210009, China; 220183453@seu.edu.cn (L.W.); 230179645@seu.edu.cn (B.W.); 101011288@seu.edu.cn (J.Z.); zhubl@jscdc.cn (B.Z.); 2 Department of Prevention and Control for Occupational Disease, Jiangsu Provincial Center for Disease Control and Prevention, Nanjing 210009, China

**Keywords:** GAPDH, SNP, NIHL, susceptibility

## Abstract

*Objective*: The purpose of this paper was to clarify the association between genetic variation in the glyceraldehyde 3-phosphate dehydrogenase (GAPDH) gene and the risk of noise-induced hearing loss (NIHL). *Methods*: A case-control study (633 cases and 625 controls) was conducted in this study. Logistic regression was used to analyze the relationships between environmental and individual factors and NIHL. Gene expression levels were compared among each *GAPDH* rs6489721 genotype and between the case and control groups based on real-time fluorescence quantitative Polymerase Chain Reaction (PCR). *Results*: The T allele of *GADPH* rs6489721 was significantly associated with NIHL (odds ratio (OR) = 1.262, 95% confidence interval (CI) (1.066, 1.493), *p* = 0.006) and showed strong associations in the codominant and dominant models (TT vs. CC: OR = 1.586, 95% CI (1.131, 2.225), *p* = 0.008; TT vs. TC/CC: OR = 1.391, 95% CI (1.073, 1.804), *p* = 0.013). The expression level of the TT genotype was significantly higher than that of the CC genotype (*p* = 0.012), and the expression of the case group was also higher than that of the control group (*p* = 0.013). *Conclusions*: The homozygous risk allele (TT) of rs6489721 was associated with an enhanced *GAPDH* expression, resulting in the development of NIHL in a Chinese population.

## 1. Introduction

Millions of workers are exposed to harmful noise in the workplace. About 12% of workers in the United States have hearing impairment, of which about 24% is considered to be caused by occupational noise exposure, with 22 million workers exposed to excessive noise [1]. About 20% of workers in Europe are also exposed to noise for >50% of their working hours [2], while more than 10 million workers in China are exposed to occupational noise, of whom about 1 million have different levels of occupational noise-induced hearing loss (NIHL) [3]. The World Health Organization estimated that 466 million people worldwide suffered from hearing loss [4]. The estimated total number of global years lived with disability (YLD) due to hearing loss of 24.9 million represents 4.7% of the total YLD due to all causes, making hearing loss the second leading cause of YLD after depression, with a larger nonfatal burden than alcohol use disorders, osteoarthritis, and schizophrenia [5]. Occupational NIHL, second only to senile deafness [6], is one of the major occupational diseases.

NIHL is a complex disease caused by the interaction of environmental and genetic factors [7,8]. Although the exact cause of NIHL is unclear, a growing number of epidemiological studies have suggested that noise, organic solvents, heavy metals, smoking, high blood pressure, and cholesterol levels are all risk factors for NIHL [9,10]. Moreover, people who work in similar noise environments may have different hearing impairments [11]. These results suggested that genetic susceptibility and its interaction with environmental factors may play important roles in the development of NIHL [6,12]. The influence of genetic factors on NIHL susceptibility has been demonstrated in animal studies. For example, glutamate peroxidase 1 (*Gpx1*) [13], heat shock factor (*Hsf1*) [14], cadherin (*Cdh23*) [15], superoxide dismutase gene 1 (*Sod1*) [16], and plasma membrane calcium ATPase isoform 2 (*Pmca2*) [17] were associated with NIHL susceptibility in knockout mice. Previous genetic studies in humans also showed that the catalase (*CAT*) [18], glutathione S transferase (*GSTM1*) [19], cadherin (*CDH23*) [20], *hOGG1* [21], *HSP70* [22], *KCNE1*, and *KCNQ4* [23] were related to the development of NIHL.

Glyceraldehyde 3-phosphate dehydrogenase (GAPDH) is a key enzyme involved in glycolysis and a housekeeping gene expressed at high levels in almost all tissues. However, increasing numbers of studies have shown that GAPDH is involved not only in energy metabolism, but also in a variety of physiological cellular functions, such as DNA repair, nuclear RNA export, maintenance of telomere structure, membrane fusion and transportation, microtubule assembly and depolymerization, cytoskeleton, cytoskeleton dynamic balance, apoptosis, and tumorigenesis [24,25]. GAPDH was expressed in cochlear tissue in newborn rats, and its expression was upregulated under deoxygenated conditions [26]. Furthermore, the conformation of GAPDH was affected by oxidative stress, leading to increased cell death [27]. GAPDH expression was also increased after nerve cell stimulation by the external environment, promoting neuronal apoptosis and leading to neurodegenerative diseases [28]. Although the mechanism of NIHL is not yet completely defined, oxidative stress is recognized as an important pathogenic factor, and its pathogenesis may be related to hair cell apoptosis induced by oxidative stress [29]. However, the relationship between the *GAPDH* gene and NIHL has not been reported. In light of the function of GAPDH in oxidative stress and its proapoptotic effect, this study aimed to explore the effect of *GAPDH* gene polymorphisms on the susceptibility to NIHL.

## 2. Subjects and Methods

### 2.1. Subjects

This was a case-control study of noise-exposed workers from the automobile, energy, and coal mining industries in Jiangsu Province, China. The labor force in these areas is highly stable and the working environments are similar, indicating that the workers remained in a stable noise environment during their work. This study was approved by the Institutional Review Board of Jiangsu Provincial Center for Disease Prevention and Control. The inclusion criteria were: (1) Han ethnicity workers with >1 year exposure to noise; (2) no history of hypertension, hyperlipidemia, otitis media or craniocerebral injury, no history of ototoxic drugs, and no history of familial hereditary deafness or blast deafness; (3) no history of fever or other common diseases such as influenza, diarrhea, pneumonia, or hepatitis for one month before the hearing examination; and (4) complete occupational health monitoring and workplace noise-detection data.

### 2.2. Methods

#### 2.2.1. Questionnaire and Physical Examination

A questionnaire to establish the individual situations of the noise-exposed workers was designed according to the following requirements: (1) general demographic information (age, sex, education level, type of work, etc.); (2) life-behavior habits, including smoking [never smoking, previous smoking (not for >3 months), smoking (at least one per day for >6 months)], drinking [(never drinking, previous drinking, drinking (at least once a week for >1 year)], sleeping, and headphone usage, etc.; (3) noise exposure (noise exposure history, exposure years) and individual protection [earplug wearing classified as non-wearing (<1 day/week), sometimes (1–2 days/week), and frequently wearing (≥3 days/week)]; and (4) current disease, previous history of otitis media, blast deafness, and familial genetic history.

#### 2.2.2. Audiological Status Assessment and Definition of NIHL

The hearing thresholds of the subjects’ left and right ears were tested at 500, 1000, 2000, 3000, 4000, and 6000 Hz, according to the requirements of the Chinese Diagnostic Criteria of Occupational NIHL (GBZ49-2014). The tests were carried out in a soundproof chamber with background noise <25 dB(A) using a Madsen Voyager 522 audiometer (Madsen, Taastrup, Denmark). All the subjects were required to have been out of the noise environment for ≥12 h before the inspection. The results were adjusted for age and sex according to GB/T7582-2004. In this study, occupational noise exposure was defined as working 8 h a day in an environment with a noise exposure level ≥85 dB(A). Individuals with a binaural high frequency (3000, 4000, 6000 Hz) average hearing threshold >25 dB(A) were classified as the NIHL group according to GBZ49-2014, and the rest as the control group. The control group was frequency-matched with the NIHL group according to age, sex, and noise exposure intensity [21].

#### 2.2.3. Single-Nucleotide Polymorphism (SNP) Selection and Genotype

Target SNPs in the *GAPDH* gene were selected based on the HapMap database and previous reports from the literature according to the following criteria: (1) detected by Haploview software; (2) minor allele frequency of CHB (Han Chinese of Beijing, China) >0.1; and (3) linkage disequilibrium r^2^ > 0.8. Four *GAPDH* SNPs (rs1136666, rs1803621, rs1060620, and rs6489721) were selected (Table 1).

DNA was extracted from peripheral blood using a QIAcube HT Plasticware and QIAamp 96 DNA QIAcube HT Kit (Qiagen, Dusseldorf, Germany) according to the manufacturer’s protocol and then stored at −80 °C in Jiangsu Provincial Center for Disease Control and Prevention until use. The genotype was determined by the Shanghai BioWing Applied Biotechnology Company (http://www.biowing.com.cn/) sing multiplex PCR with next generation sequencing [30]. Targets were initially amplified by a 10 μL PCR reaction containing 2 μL human DNA, 0.1 μL of Hot Start DNA Polymerase (Biowing biotechnology, Shanghai, China), 1 μL PCR buffer, 0.8 μL dNTPs, 1 μL MgSO4, 3.2 μL ddH2O, 10 μL paraffin oil, and 2 μL of each specific primer in the first round PCR. The following cycling programs were used for the PCR: 95 °C for 15 min, 4 cycles of [94 °C for 30 s, 60 °C for 10 min, 72 °C for 30 s], 20 cycles of [94 °C for 30 s, 60 °C for 1 min, 72 °C for 30 s]. The second round of 10 μL PCR reaction used a 3 μL template which was the first-round PCR products, 0.1 μL of Hot Start DNA Polymerase (Biowing biotechnology, Shanghai, China), 1 μL PCR buffer, 0.8 μL dNTPs, 1 μL MgSO4, 3.2 μL ddH2O, and 10 μL paraffin oil. The following cycling programs were used for the PCR: 95 °C for 15 min, 4 cycles of [94 °C for 30 s, 60 °C for 10 min, 72 °C for 30 s], and 40 cycles of [94 °C for 30 s, 65 °C for 1 min, 72 °C for 30 s]. The third-round PCR involved adding into the second-round PCR tube a 10 μL reaction mixture which contained 0.1 μL of Hot Start DNA Polymerase (Biowing biotechnology, Shanghai, China), 2 μL PCR buffer, 0.8 μL dNTPs, 1 μL MgSO4, 3.6 μL ddH2O, 10 μL paraffin oil, and 3.6 μL Barcode. The following cycling programs were used for the PCR: 95 °C for 15 min, 4 cycles of [94 °C for 30 s, 60 °C for 4 min, 72 °C for 30 s], 40 cycles of [94 °C for 30 s, 65 °C for 1 min, 72 °C for 30 s]. The bridge PCR products were sequenced on the Illumina X-10 (Illumina Technologies Corporation, California, American) sequencing platform, and the operation flow was carried out according to the standard SOP. The primers used for genotyping are listed in Table 1.

Total RNA was extracted from whole blood using TRIzol, and cDNA was then obtained by reverse transcription reaction with 5× PrimeScript RT Master mix (Takara, Japan). A real-time fluorescence quantitative PCR was performed using SYBR^®^ Green Realtime PCR Master Mix (Takara) according to the following procedures: denaturation at 95 °C for 30 s, followed by 45 cycles of denaturation at 95 °C for 10 s, annealing at 60 °C for 30 s, and extension at 72 °C for 10 s. The expression of the *GAPDH* gene was calculated by the 2^−ΔΔCt^ method. The primer sequences of the *GAPDH* and β-actin genes were as follows (5′~3′): GAPDH-forward: GGACCTGACCTGCCGTCTAG, GAPDH-reverse: GTAGCCCAGGATGCCCTTGA; and β-actin-forward: CTACCTCATGAAGATCCTCACCGA, β-actin-reverse: TTCTCCTTAATGTCACGCACGATT.

#### 2.2.4. Statistical Analysis

The statistical analysis was performed using SPSS 25.0 software. The Hardy-Weinberg equilibrium test was carried out with a goodness of fit χ^2^ test among the control subjects. The continuous variables were analyzed by Student’s *t*-test and the categorical variables by Pearson’s χ^2^ test. A binary logistic regression model was used to correct for confounding factors such as age, sex, smoking, and drinking status, and to analyze the relationships between environmental and individual factors and NIHL. The potential gene-environment interaction was detected by stratified and crossover analyses. Odds ratios (ORs) and 95% confidence intervals (CIs) were used to indicate the intensity of association, and a one-way ANOVA was used to compare the average high-frequency hearing thresholds between individuals with each genotype at each SNP. The interaction between the SNPs was tested by a multifactor dimensionality reduction (MDR) analysis. The haplotype analysis of the polymorphisms was performed using the SHEsis platform [31]. The patterns were characterized using Haploview 4.1 software. The haplotype *p* value was corrected using the Sidak Holm’s correction, with *p* < 0.05 as the cutoff for statistical significance. The level of significance was set at a = 0.05.

## 3. Results

### 3.1. Case and Control Groups

**Figure 1:**
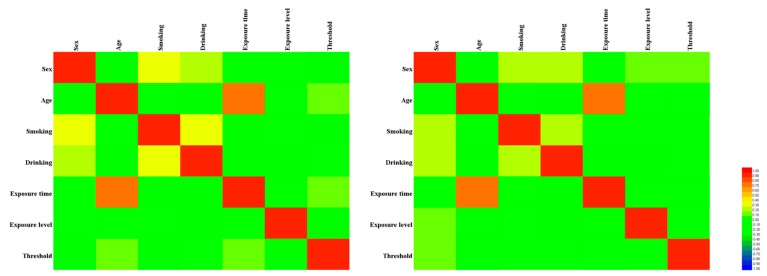
Heatmap based on the correlation between factors such as sex, age, smoking, drinking, exposure time, exposure level, and threshold shift. The left is the case group and the right is the control group.

A total of 1258 individuals were included in the study, including 633 in the case group and 625 in the control group (Table 2). There were no significant differences between the groups in terms of age, sex, noise exposure level, noise exposure time, smoking, and drinking status (all *p* > 0.05). The distributions of these factors between the two groups were balanced and comparable. However, the average hearing threshold was significantly higher in the case compared with the control group (*p* < 0.001). As can be seen from Figure 1, regardless of the case group or the control group, only the age and exposure time showed a strong correlation (|r| > 0.5), and other factors were moderately or weakly correlated [32], which suggests that perhaps genetic factors play an important role in hearing loss.

### 3.2. Selected SNPs

Four SNPs (rs1136666, rs1803621, rs1060620, and rs6489721) were selected according to the screening criteria (Table 3). The Hardy-Weinberg equilibrium test *p* value was >0.05, indicating that all the SNPs met the Hardy-Weinberg equilibrium in the control group.

### 3.3. Relationship between GAPDH Gene Polymorphisms and NIHL Susceptibility Under Different Gene Models

The genotypes and allele distributions of *GAPDH* in 1258 subjects (633 cases and 625 controls) are shown in Table 4. After adjusting for age, sex, smoking, and drinking, the binary logistic regression analysis showed that the risk of NIHL in CC carriers in the rs1136666 dominant model was 1.259 times higher than that in CG/GG carriers (95% CI (1.002, 1.583), *p* = 0.048). The risk of NIHL in TT carriers in the codominant model of rs6489721 was 1.586 times higher than that in CC carriers (95% CI (1.131, 2.225), *p* = 0.008). In the dominant model, TT carriers had a 1.391 times higher risk of NIHL than TC/CC carriers (95% CI (1.073, 1.804), *p* = 0.013), and in the allele model the risk of NIHL was 1.262 times higher in individuals with the T allele compared with the C allele (95% CI (1.066, 1.493), *p* = 0.006). The results suggest that the *GAPDH* SNPs rs1136666 and rs6489721 may be related to NIHL and rs6489721TT is a risk genotype.

### 3.4. Stratified Analyses of rs1136666, rs1803621, rs1060620, and rs6489721 Polymorphisms

The influences of the rs1136666, rs1803621, rs1060620, and rs6489721 genotypes on a series of risk characteristics for NIHL were examined in a dominant model (Table 5). For rs1136666, there was an increased risk of NIHL in subjects with the CC compared with the CG/GG genotype at exposure levels ≤85 dB(A) (OR = 1.511, 95% CI (1.011, 2.258), *p* = 0.044), which may be due to the fact that the role of the gene is relatively strong with a lower exposure level ≤85 dB, but when the noise exposure intensity is too high the noise plays a major role and the effect of the gene is too weak to be observed, which suggests that noise intensity is still a major NIHL risk factor. In men, the genotype distributions of rs1060620 and rs6489721 were significantly different between the case and control groups (*p* = 0.027 and 0.011, respectively), and the AG/AA genotype of rs1060620 and TT genotype of rs6489721 were associated with increased risks of NIHL (OR = 1.356, 95% CI (1.035, 1.778); OR = 1.419, 95% CI (1.085, 1.855), respectively). Moreover, there were significant differences in the genotype distributions for rs6489721 between the case and control groups among individuals with an exposure time ≤20 years (*p* = 0.016, OR = 1.501, 95% CI (1.079, 2.088)), never smokers (*p* = 0.009, OR = 1.711, 95% CI (1.138, 2.571)), and never drinkers (*p* = 0.004, OR = 1.641, 95% CI: (1.166, 2.309)), and the TT genotype was identified as a risk genotype.

To further explore the interaction between genes and other factors, we conducted a crossover analysis of rs6489721 with the age and noise exposure levels, respectively. The results are shown in Table 6. Compared with individuals with wild TT genotypes and noise exposure levels ≤85 dB(A), individuals with mutant genotypes (TC + CC) and noise exposure levels >92 dB(A) had a significantly reduced risk of NIHL (OR = 0.495, 95% CI (0.311, 0.790)), indicating an interaction between the genotype and noise exposure level (*p* = 0.003). It can also be concluded that there was no statistically significant interaction between rs6489721 and age (*p* > 0.05).

### 3.5. Associations between Haplotypes of GAPDH SNPs and NIHL Risk

The results of the haplotype analysis are shown in Table 7. Four common haplotypes (frequency >3%) were selected from 99% of the haplotype variants derived from four SNPs, and the rest were included in the mixed group. Haplotype rs1136666G-rs1803621C-rs1060620G-rs6489721C (OR = 0.791, 95% CI (0.658, 0.952), *p* = 0.013) was a protective factor for NIHL, and no haplotype increased the risk of developing NIHL.

### 3.6. Average Hearing Thresholds in Relation to the Genotypes of Four SNPs

**Figure 2:**
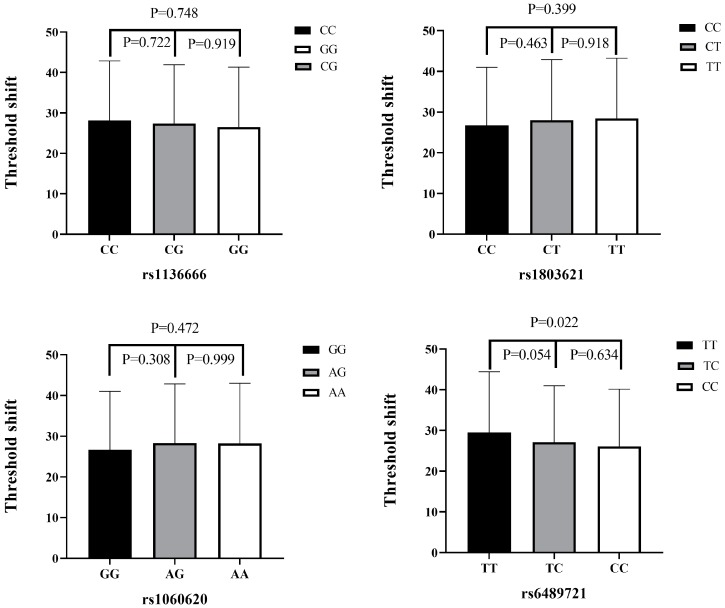
High-frequency hearing threshold shifts according to SNP genotype. A comparison of high-frequency hearing threshold shifts among rs1136666, rs1803621, rs1060620, and rs6489721 genotypes in all subjects. Data presented as mean ± standard deviation and analyzed by ANOVA.

The high-frequency hearing threshold shifts of the four SNPs (rs1136666, rs1803621, rs1060620, and rs6489721) are shown in Figure 2. For rs6489721, the TT genotype was mainly in the range of 29.51 ± 14.94 dB, while the CC genotype was in the range of 26.09 ± 14.04 dB. The high-frequency hearing threshold shift in rs6489721 TT genotype individuals was significantly higher than in CC genotype individuals (*p* = 0.022). Apparently, the risk genotype TT is associated with a higher hearing threshold shift. There was no difference in the high-frequency hearing threshold shift between the genotypes at other sites (*p* > 0.05).

### 3.7. MDR Analysis of Interactions among the Four SNPs

**Figure 3:**
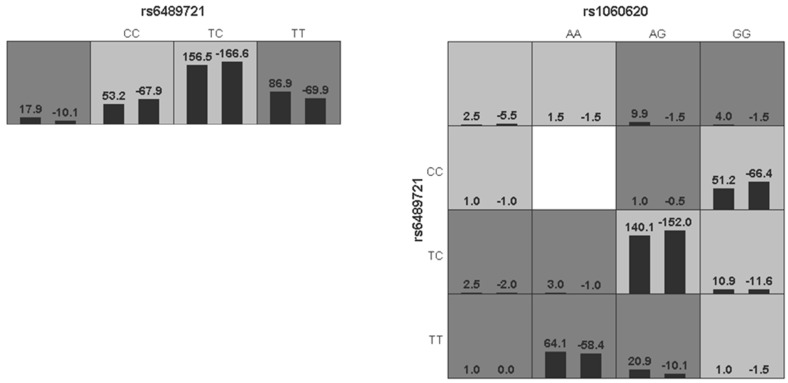

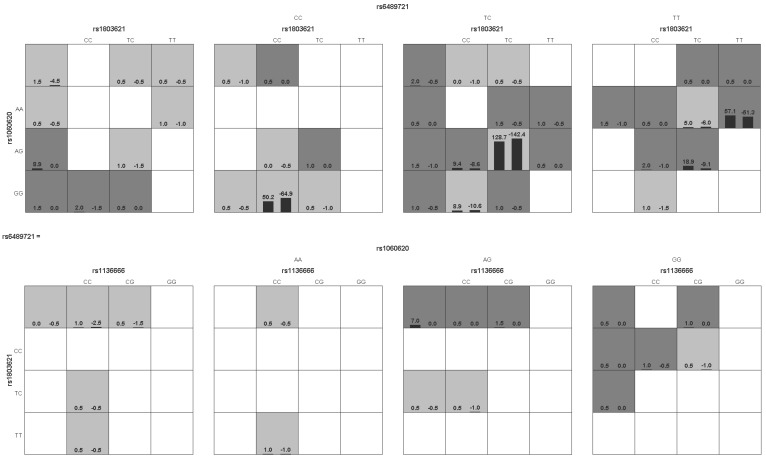

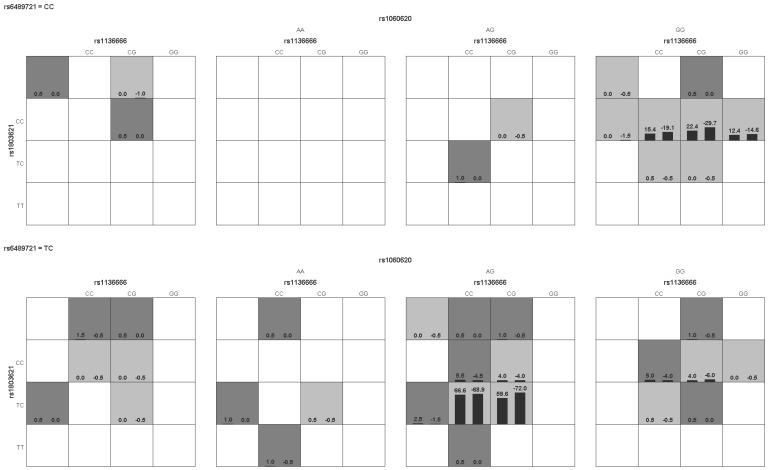

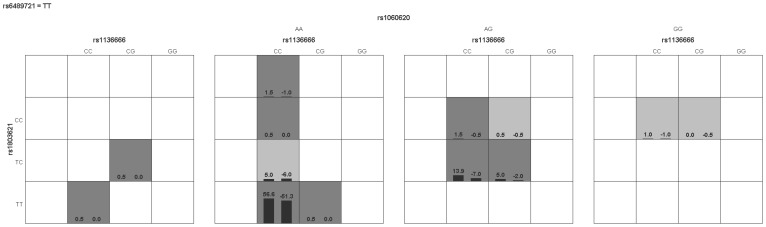
Interactions among the four SNPs. The dark and light gray boxes represent high and low risk factor combinations, respectively. The left bars within each box represent cases and the right bars represent controls. The heights of the bars are proportional to the sum of samples in each group.

The results of the MDR analysis of the four SNP interactions are shown in Table 8 and Figure 3. There were no interactions among these SNPs (*p* > 0.05). The optimal model indicated that rs6489721 acting alone increased the risk of NIHL (*p* < 0.05).

### 3.8. GAPDH Gene Expression in Relation to rs6489721 Genotype and Presence of NIHL

**Figure 4:**
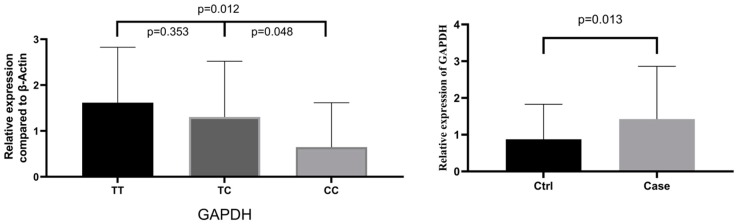
Relative *GAPDH* expression levels according to the rs6489721 genotype and presence of NIHL.

*GAPDH* gene expression was significantly higher in individuals with the rs6489721 TT or TC genotype compared with the CC genotype (*p* = 0.012 and 0.048, respectively) (Figure 4). *GAPDH* gene expression was also significantly higher in the cases compared with the controls (*p* = 0.013). These results suggest that a high level of *GAPDH* gene expression is a risk factor for NIHL.

## 4. Discussion

The results of the current study demonstrated an association between the *GAPDH* gene and NIHL. The rs6489721-T allele was associated with an increased risk of NIHL, and a higher expression of *GAPDH* also increased the risk of NIHL. Research into NIHL susceptibility genes and SNPs has been increasing, from 170 related publications in 2009 to 249 in 2018. Several NIHL-related susceptibility genes are involved in oxidative stress pathways, potassium cycle pathways, heat shock protein genes, calcineurin genes, Notch signaling pathways, apoptotic signaling pathways, and NIHL-related single genes [33].

Previous studies have shown that noise can cause hypoxia and energy metabolism disorders in cochlear hair cells, leading to hair-cell death [34]. The death of hair cells occurs via two distinct pathways, apoptosis and necrosis, with apoptosis being the main pathway [35]. Mitochondria act as an important cellular control center via their roles in the cellular respiratory chain and oxidative phosphorylation, and also in apoptosis. Mitochondria are the main sites of intracellular energy metabolism and the production of reactive oxygen species (ROS) and free radicals [36]. Noise exposure increases the energy requirements of cochlear hair cells leading to increased mitochondrial activities and the consequent release of free radical products. Increased oxygen free radicals in the mitochondria of the cochlear hair cells in turn leads to DNA damage, which causes the release of the apoptosis-inducing factor from the mitochondria into the cytoplasm, activating caspase-3 and triggering apoptosis [37].

In addition to acting as an internal reference gene, GAPDH is known to be located in the cytoplasm, nucleus, and cell membrane and to be regulated at the transcription and translation levels, leading to changes in mRNA and protein levels in response to various stimuli. GAPDH interacts with tRNA to form a GAPDH/tRNA complex that promotes tRNA transport out of the nucleus. NAD+ competitively binds to GAPDH to inhibit complex formation and tRNA nucleation [38]. In addition, mutagens can convert cytosine into uracil by deamination, which is usually repaired by uracil DNA glycosylase. GAPDH has uracil DNA glycosylase activity that can remove the mutated uracil [39], thus implicating GAPDH in DNA repair. Early reports identified the membrane-integration characteristic of GAPDH [40] and subsequently observed its important role in endocytosis and nuclear membrane assembly. In this study, we examined the genetic associations of four *GAPDH* SNPs (rs1136666, rs1803621, rs1060620, and rs6489721) in 1258 noise-exposed subjects (633 cases, 625 control) in relation to susceptibility to NIHL. The SNPs rs1136666 and rs6489721 were potentially related to NIHL susceptibility. Notably, rs6489721 was significantly associated with NIHL; the T-allele frequency was higher in cases than in controls, suggesting that the rs6489721-T allele was associated with an increased risk of NIHL and that the TT genotype was a risk genotype. By a stratified analysis, we found that the risk of hearing loss in rs1136666CC genotypes increased with a lower exposure level ≤85 dB and was absent at higher levels, which suggests that noise intensity is still a major NIHL risk factor. A further crossover analysis showed an interaction between rs6489721 genotypes and noise exposure levels. This result suggests that in the general population without the rs6489721 mutant allele as a protective factor, the prevention of NIHL should pay more attention to noise exposure intensity, so as to achieve individualized prevention and treatment. A subsequent haplotype analysis showed that the rs1136666G-rs1803621C-rs1060620G-rs6489721C haplotype reduced NIHL susceptibility. Importantly, individuals with the TT genotype had a significantly higher average high-frequency hearing threshold than individuals with the CC genotype.

Recent studies showed that GAPDH plays an important role in apoptosis. Apoptosis was increased in an overexpression model of cells transfected with *GAPDH* cDNA [41]. The role of *GAPDH* in Parkinson’s disease has also caused wide attention. Anti-dementia protective drugs such as the cholinesterase inhibitors tacrine, donezepil, and selegiline, as well as selective monoamine oxidase B inhibitors, are widely used for the treatment of Parkinson’s disease, mainly relying on their ability to interact with the GAPDH apoptotic cascade [42]. In our study, the expression levels of the rs6489721 TT genotype *GAPDH* gene were significantly higher than those of the CC genotype, and the *GAPDH* gene expression levels were also significantly higher in the cases compared with the controls, suggesting that an increased expression of *GAPDH* and the rs6489721 TT genotype were associated with an increased risk of NIHL. These results supported our hypothesis that *GAPDH* polymorphisms were associated with NIHL susceptibility, and that an increased gene expression of *GAPDH* increased the risk of NIHL.

GAPDH is an oxidation-sensitive protease, and the oxidation of Cys152 under conditions of mitochondrial dysfunction and elevated ROS levels may result in the inhibition of GAPDH, which is involved in cell death. On the other hand, GAPDH expression in mitochondria increases sharply during oxidative stress, and it can regulate mitochondrial membrane permeability through voltage-dependent ion channels, reduce the mitochondrial transmembrane voltage, expand the matrix, promote the release of cytochrome C and other pro-apoptotic proteins from the mitochondria, and further increase the level of cellular oxidative stress, thus promoting apoptosis [43]. *GAPDH* was shown to be expressed in the cochlea of mice, and its expression was upregulated during oxidative stress [26]. The possible mechanism by which GAPDH increases the risk of NIHL is as follows: Noise exposure leads to an increase in ROS in mitochondria, leading to apoptosis. However, the rise in ROS also increases GAPDH expression [26], which is inserted into the nucleus through the guidance of the E3 common ligase Siah1, and p53 is then activated through P300/CREB acetylation, thus promoting apoptosis [44]. Conversely, the increased GAPDH expression can aggravate ROS and promote apoptosis [43]. In addition, *GAPDH* gene mutation can lead to its increased expression, such as in rs6489721-TT genotype individuals, promoting apoptosis and making individuals with this genotype at a higher risk of developing NIHL.

## 5. Conclusions

This study provides the first evidence of an association between the rs6489721 SNP and enhanced *GAPDH* expression, contributing to the development of NIHL in a Chinese Han population. These results suggested that genetic variation in the *GAPDH* gene may act as a biomarker of genetic susceptibility to NIHL. The interaction between genetic variation and noise exposure may play an important role in the pathogenesis of NIHL; however, further research is needed to explore the molecular mechanisms of this interaction. Given the widespread presence of GAPDH throughout the body, identifying its association with NIHL may contribute to developing more effective prophylactic and therapeutic methods for NIHL, with beneficial implications for the health of noise-exposed workers.

## Figures and Tables

**Table 1:** Primers for the SNP markers.

SNP ID	Forward Primer (5′~3′)	Reverse Primer (5′~3′)
rs1136666	CTAGGCGCTCACTGTTCTC	CTGACCTTGAGCTCTCCTTG
rs1803621	GAAAAACCTGCCAAATATGATGAC	GCACTTTTTAAGAGCCAGTCTC
rs1060620	TTTATGGAGGTCCTCTTGTGTC	CATTTACAGCCTGGCCTTTG
rs6489721	TCTCAGCCTTTGAAAGAAAGAAAG	GAAGGGACTGAGATTGGCC

**Table 2:** Demographic characteristics of the study subjects.

Variables	Case (n = 633)		Control (n = 625)		*p*
	n	%	n	%	
Age (years) (Mean ± SD)	40.2 ± 7.4		40.2 ± 7.9		0.989 *
Sex					
Male	593	93.7	587	93.9	0.860 **
Female	40	6.3	38	6.1	
Exposure level [dB(A)]	87.4 ± 7.7		88.2 ± 7.8		0.101 *
Exposure time (years)	18.3 ± 8.4		17.6 ± 8.8		0.109 *
Threshold [dB(A)]	37.5 ± 12.1		15.3 ± 4.7		<0.001 *
Smoking					
Now	371	58.6	351	56.2	0.675 **
Ever	13	2.1	13	2.1	
Never	249	39.3	261	41.8	
Drinking					
Now	272	43.0	272	43.5	0.967 **
Ever	11	1.7	10	1.6	
Never	350	55.3	343	54.9	

* Student’s *t*-test; ** Pearson’s χ^2^ test. The bold in this table means *p* value < 0.05.

**Table 3:** Selected SNPs and Hardy-Weinberg test.

SNP	Alleles	Chromosome	TagSNPs	MAF	*p* for HWE ^b^
				HapMap ^a^	
rs1136666	C/G	12:6534825	rs1136666	0.25 (G)	0.305
rs1803621	C/T	12:6537943	rs1060620	0.41 (C)	0.159
rs1060620	G/A	12:6535556	rs1060620; rs1803621; rs1065691; rs2886093; rs1060619; rs1803622	0.41 (G)	0.165
rs6489721	T/C	12:6534150	rs3741918	0.34 (C)	0.068

^a^ MAF from the HapMap database (http://www.hapmap.org). ^b^ *p* value for the Hardy-Weinberg test.

**Table 4:** Distribution of four SNPs and associations with NIHL.

Genetic Model	Genotypes	Case		Control		*p* ^a^	Adjusted OR 95% (CI) ^a^
		n = 633		n = 625			
**rs1136666**		n = 606	%	n = 615	%		
Codominant	CC	371	61.2	343	55.7	0.343	1.305 (0.752,2.266)
	CG	210	34.6	242	39.3	0.886	1.042 (0.594,1.829)
	GG	25	4.1	30	4.8		1.000 (Reference)
Dominant	CC	371	61.2	343	55.7	**0.048**	**1.259 (1.002,1.583)**
	CG/GG	235	38.7	272	44.1		1.000 (Reference)
Recessive	CG/CC	581	95.8	585	95	0.517	1.197 (0.695,2.062)
	GG	25	4.1	30	4.8		1.000 (Reference)
Alleles	C	952	78.5	928	75.4		1.000 (Reference)
	G	260	21.5	302	24.6	0.059	0.830 (0.684,1.007)
rs1803621		n = 593	%	n = 606	%		
Codominant	CC	150	25.2	178	29.3		1.000 (Reference)
	TC	321	54.1	322	53.1	0.222	1.181 (0.904,1.543)
	TT	122	20.5	106	17.4	0.080	1.354 (0.964,1.902)
Dominant	CC	150	25.2	178	29.3		1.000 (Reference)
	TC/TT	443	74.6	428	70.5	0.120	1.224 (0.949,1.580)
Recessive	TC/CC	471	79.3	500	82.4		1.000 (Reference)
	TT	122	20.5	106	17.4	0.192	1.213 (0.908,1.621)
Alleles	C	621	52.4	678	55.9		1.000 (Reference)
	T	565	47.6	534	44.1	0.073	1.166 (0.986,1.380)
rs1060620		n = 619	%	n = 608	%		
Codominant	GG	135	21.8	161	26.4		1.000 (Reference)
	AG	346	55.8	326	53.6	0.093	1.265 (0.961,1.664)
	AA	138	22.2	121	19.9	0.076	1.355 (0.969,1.894)
Dominant	GG	135	21.8	161	26.4		1.000 (Reference)
	AG/AA	484	78.0	447	73.5	0.058	1.289 (0.991,1.676)
Recessive	AG/GG	481	77.6	487	80.0		1.000 (Reference)
	AA	138	22.2	121	19.9	0.318	1.150 (0.874,1.515)
Alleles	G	616	49.8	648	53.3		1.000 (Reference)
	A	622	50.2	568	46.7	0.070	1.167 (0.987,1.379)
rs6489721		n = 597	%	n = 605	%		
Codominant	TT	175	29.3	139	22.9	**0.008**	**1.586 (1.131,2.225)**
	TC	315	52.7	331	54.7	0.234	1.198 (0.890,1.612)
	CC	107	17.9	135	22.3		1.000 (Reference)
Dominant	TT	175	29.3	139	22.9	**0.013**	**1.391 (1.073,1.804)**
	TC/CC	422	70.6	466	77.0		1.000 (Reference)
Recessive	TC/TT	490	82.0	470	77.6	0.061	1.312 (0.988,1.743)
	CC	107	17.9	135	22.3		1.000 (Reference)
Alleles	T	665	55.7	609	50.3	**0.006**	**1.262 (1.066,1.493)**
	C	529	44.3	601	49.7		1.000 (Reference)

^a^ Adjusted for age, sex, smoking, drinking in logistic regression model. The bold in this table means *p* value < 0.05.

**Table 5:** Stratified analyses of SNPs in a dominant model.

**rs1136666**						
**Variables**	**CC (case/control)**		**CG/GG (case/control)**		***p*^a^**	**OR (95% CI) ^a^**
	**n**	**%**	**n**	**%**		
Age (years)						
<40	161/142	30.1/26.5	108/124	20.2/23.2	0.131	1.302 (0.924,1.834)
≥40	210/201	30.6/29.3	127/148	18.5/21.6	0.207	1.218 (0.897,1.653)
Sex						
Male	344/322	30.1/28.1	223/255	19.5/22.3	0.095	1.222 (0.965,1.546)
Female	27/21	35.1/27.3	12/17	15.6/22.1	0.206	1.821 (0.716,4.632)
Exposure level [dB(A)]						
≤85	142/94	35.5/23.5	82/82	20.5/20.5	**0.044**	**1.511 (1.011,2.258)**
85–92	65/54	31.9/26.5	43/42	21.1/20.6	0.569	1.176 (0.673,2.054)
>92	112/119	28.6/30.4	75/85	19.2/21.7	0.754	1.067 (0.712,1.597)
Exposure time (years)						
≤20	219/211	29.6/28.5	138/173	18.6/23.3	0.078	1.301 (0.971,1.744)
>20	152/132	31.7/27.5	97/99	20.2/20.6	0.385	1.175 (0.816,1.692)
Smoking						
Now	215/186	30.6/26.5	142/160	20.2/22.8	0.083	1.302 (0.966,1.757)
Ever	5/8	20.8/33.3	6/5	25.0/20.8	0.431	0.521 (0.102,2.658)
Never	151/149	30.6/30.2	87/107	17.6/21.7	0.233	1.246 (0.868,1.791)
Drinking						
Now	152/148	28.9/28.1	106/120	20.2/22.8	0.393	1.163 (0.823,1.643)
Ever	8/3	38.1/14.3	3/7	14.3/33.3	0.086	6.222 (0.936,41.382)
Never	211/192	31.3/28.5	126/145	18.7/21.5	0.136	1.265 (0.929,1.722)
rs1803621						
Variables	CC (case/control)		TC/TT (case/control)		*p* ^a^	OR (95% CI) ^a^
	n	%	n	%		
Age (years)						
<40	62/72	11.9/13.8	200/189	38.2/36.1	0.304	0.814 (0.549,1.206)
≥40	88/106	13.0/15.7	243/239	35.9/35.4	0.234	0.817 (0.584,1.141)
Sex						
Male	139/171	12.4/15.2	414/398	36.9/35.5	0.066	0.781 (0.601,1.016)
Female	11/7	14.3/9.1	29/30	37.7/39.0	0.374	1.626 (0.554,4.769)
Exposure level [dB(A)]						
≤85	50/52	12.7/13.2	171/122	43.3/30.9	0.102	0.686 (0.436,1.078)
85–92	33/29	16.5/14.5	74/64	37.0/32.0	0.958	0.984 (0.540,1.794)
>92	43/48	11.3/12.7	136/152	35.9/40.1	0.996	1.001 (0.624,1.605)
Exposure time (years)						
≤20	91/105	12.5/14.4	260/273	35.7/37.4	0.573	0.910 (0.655,1.263)
>20	59/73	12.6/15.5	183/155	38.9/33.0	0.066	0.685 (0.457,1.026)
Smoking						
Now	90/100	13.1/14.6	256/240	37.3/35.0	0.320	0.844 (0.604,1.179)
Ever	3/2	12.0/8.0	9/11	36.0/44.0	0.645	1.833 (0.250,13.470)
Never	57/76	11.7/15.6	178/177	36.5/36.3	0.152	0.746 (0.499,1.114)
Drinking						
Now	66/79	12.9/15.4	187/180	36.5/35.2	0.268	0.804 (0.547,1.183)
Ever	4/3	20.0/15.0	6/7	30.0/35.0	1.000	1.556 (0.244,9.913)
Never	80/96	12.0/14.4	250/241	37.5/36.1	0.214	0.803 (0.569,1.135)
rs1060620						
Variables	GG (case/control)		AG/AA (case/control)		*P* ^a^	OR (95% CI) ^a^
	n	%	n	%		
Age (years)						
<40	58/71	10.7/13.1	217/194	40.2/35.9	0.120	0.730 (0.491,1.087)
≥40	77/90	11.2/13.1	267/253	38.9/36.8	0.239	0.811 (0.572,1.150)
Sex						
Male	125/155	10.9/13.5	455/416	39.5/36.1	**0.027**	**1.356 (1.035,1.778)**
Female	10/6	13.2/7.9	29/31	38.2/40.8	0.314	1.782 (0.575,5.525)
Exposure level [dB(A)]						
≤85	44/47	11.1/11.8	181/125	45.6/31.5	0.068	0.647 (0.404,1.035)
85–92	27/25	13.2/12.2	83/70	40.5/34.1	0.771	0.911 (0.485,1.710)
>92	44/43	11.0/10.8	154/159	38.5/39.8	0.821	1.056 (0.657,1.699)
Exposure time (years)						
≤20	82/99	11.0/13.2	284/283	38.0/37.8	0.262	0.825 (0.590,1.155)
>20	53/62	11.1/12.9	200/164	41.8/34.2	0.097	0.701 (0.460,1.068)
Smoking						
Now	80/91	11.4/12.9	283/249	40.3/35.4	0.144	0.774 (0.548,1.093)
Ever	1/1	4.0/4.0	12/11	48.0/44.0	1.000	0.917 (0.051,16.494)
Never	54/69	10.8/13.8	189/187	37.9/37.5	0.220	0.774 (0.514,1.166)
Drinking						
Now	61/72	11.6/13.6	206/189	39.0/35.8	0.210	0.777 (0.524,1.153)
Ever	1/3	4.8/14.3	10/7	47.6/33.3	0.311	0.233 (0.020,2.733)
Never	73/86	10.8/12.7	268/251	39.5/37.0	0.206	0.795 (0.557,1.135)
rs6489721						
Variables	TT (case/control)		TC/CC (case/control)		*p* ^a^	OR (95% CI) ^a^
	n	%	n	%		
Age (years)						
<40	76/58	14.4/11.0	188/206	35.6/39.0	0.072	1.436 (0.967,2.131)
≥40	99/81	14.7/12.0	234/260	34.7/38.6	0.080	1.358 (0.964,1.913)
Sex						
Male	163/128	14.5/11.4	395/440	35.1/39.1	**0.011**	**1.419 (1.085,1.855)**
Female	12/11	15.8/14.5	27/26	35.5/34.2	0.921	1.051 (0.394,2.798)
Exposure level [dB(A)]						
≤85	64/40	16.5/10.3	154/129	19.8/33.3	0.211	1.340 (0.847,2.121)
85–92	35/22	17.4/10.9	73/71	36.3/35.3	0.170	1.547 (0.828,2.892)
>92	55/48	14.1/12.3	130/156	33.4/40.1	0.166	1.375 (0.875,2.160)
Exposure time (years)						
≤20	108/86	14.7/11.7	246/294	33.5/40.1	**0.016**	**1.501 (1.079,2.088)**
>20	67/53	14.3/11.3	176/172	37.6/36.8	0.320	1.235 (0.814,1.875)
Smoking						
Now	98/82	14.2/11.9	252/256	36.6/37.2	0.265	1.214 (0.863,1.707)
Ever	3/3	12.5/12.5	9/9	37.5/37.5	1.000	1.000 (0.158,6.346)
Never	74/54	15.1/11.0	161/201	32.9/41.0	**0.009**	**1.711 (1.138,2.571)**
Drinking						
Now	61/59	11.8/11.4	195/202	37.7/39.1	0.742	1.071 (0.712,1.611)
Ever	4/2	19.0/9.5	7/8	33.3/38.1	0.635	2.286 (0.316,16.512)
Never	110/78	16.6/11.7	220/256	33.1/38.6	**0.004**	**1.641 (1.166,2.309)**

^a^ Two-sided χ^2^ test. The bold in this table means *p* value < 0.05.

**Table 6:** Crossover analysis of the interaction between rs6489721 and other factors on NIHL risk.

Factors	Genotype	Case (N)	%	Control (N)	%	*p* ^a^	OR (CI95%) ^a^
Age							
<40	TT	76	12.7	58	9.6		1.0
<40	TC + CC	188	31.5	206	34.0	0.077	0.700 (0.472–1.040)
≥40	TT	99	16.6	81	13.4	0.728	0.923 (0.588–1.450)
≥40	TC + CC	234	39.2	260	43.0	0.050	0.679 (0.462–1.000)
Exposure level [dB(A)]							
≤85	TT	64	12.5	40	8.6		1.0
≤85	TC + CC	154	30.1	129	27.7	0.205	0.742 (0.469–1.177)
85–92	TT	35	6.8	22	4.7	0.939	0.974 (0.501–1.896)
85–92	TC + CC	73	14.3	71	15.2	0.075	0.627 (0.374–1.049)
>92	TT	55	10.8	48	10.3	0.171	0.676 (0.386–1.184)
>92	TC + CC	130	25.4	156	33.5	**0.003**	**0.495 (0.311** **–** **0.790)**

^a^ Adjusted for age, sex, smoking, drinking in the logistic regression model. The bold in this table means *p* value <0.05.

**Table 7:** Frequencies of inferred haplotypes in cases and controls and their associations with NIHL risk.

Haplotypes ^a^	Case (n = 566)		Control (n = 566)		*p* ^b^	Adjusted OR (95% CI) ^c^	Global *p* ^d^	Holm	SidakSS	SidakSD
	N	%	N	%			0.058			
CCGC	266	23.2	291	24.7	0.170	0.876 [0.726–1.058]		0.511	0.893	0.429
CTAT	535	46.8	516	43.9	0.618	1.041 [0.888–1.219]		0.618	0.999	0.618
GCGC	232	20.3	283	24.1	**0.007**	**0.766 [0.631–0.931]**		0.029	0.084	0.028
CCGT	53	4.6	40	3.4	0.189	1.321 [0.870–2.007]		0.511	0.919	0.429

^a^ Alleles of haplotypes were arrayed as rs1136666-rs1803621-rs1060620-rs6489721. ^b^ Two-sided χ^2^ test. ^c^ Adjusted for age, sex, smoking, and drinking in the logistic regression model. ^d^ Generated by a permutation test with 1000 simulations. Global result: total controls = 625, total cases = 633; global χ^2^ = 7.462, Pearson’s *p* = 0.058. The bold in this table means *p* value <0.05.

**Table 8:** MDR analysis of the interactions among the four SNPs.

Model	Training Bal.ACC	Testing Bal.Acc	CV Consistency	Sign Test (*p*)
rs6489721	0.5395	0.5394	10/10	9 (0.0107)
rs1060620-rs6489721	0.5512	0.5155	8/10	7 (0.1719)
rs1803621-rs1060620-rs6489721	0.5611	0.5198	10/10	7 (0.1719)
rs1136666-1803621-rs1060620-rs6489721	0.5690	0.5143	10/10	7 (0.1719)
